# Targeting G9a/DNMT1 methyltransferase activity impedes IGF2-mediated survival in hepatoblastoma

**DOI:** 10.1097/HC9.0000000000000378

**Published:** 2024-01-29

**Authors:** Salih Demir, Negin Razizadeh, Emilie Indersie, Sophie Branchereau, Stefano Cairo, Roland Kappler

**Affiliations:** 1 Department of Pediatric Surgery, Dr. von Hauner Children’s Hospital, LMU University Hospital, LMU Munich, Germany; 2 XenTech, Evry, France; 3 Department of Pediatric Surgery, Bicêtre Hospital, AP-HP Paris Saclay University, France; 4 Champions Oncology, Inc., Rockville, Maryland, USA

## Abstract

**Background:**

As the variable clinical outcome of patients with hepatoblastoma (HB) cannot be explained by genetics alone, the identification of drugs with the potential to effectively reverse epigenetic alterations is a promising approach to overcome poor therapy response. The gene *ubiquitin like with PHD and ring finger domains 1* (UHRF1) represents an encouraging epigenetic target due to its regulatory function in both DNA methylation and histone modifications and its clinical relevance in HB.

**Methods:**

Patient-derived xenograft *in vitro* and *in vivo* models were used to study drug response. The mechanistic basis of CM-272 treatment was elucidated using RNA sequencing and western blot experiments.

**Results:**

We validated in comprehensive data sets that UHRF1 is highly expressed in HB and associated with poor outcomes. The simultaneous pharmacological targeting of UHRF1-dependent DNA methylation and histone H3 methylation by the dual inhibitor CM-272 identified a selective impact on HB patient-derived xenograft cell viability while leaving healthy fibroblasts unaffected. RNA sequencing revealed downregulation of the IGF2-activated survival pathway as the main mode of action of CM-272 treatment, subsequently leading to loss of proliferation, hindered colony formation capability, reduced spheroid growth, decreased migration potential, and ultimately, induction of apoptosis in HB cells. Importantly, drug response depended on the level of IGF2 expression, and combination assays showed a strong synergistic effect of CM-272 with cisplatin. Preclinical testing of CM-272 in a transplanted patient-derived xenograft model proved its efficacy but also uncovered side effects presumably caused by its strong antitumor effect in IGF2-driven tumors.

**Conclusions:**

The inhibition of UHRF1-associated epigenetic traces, such as IGF2-mediated survival, is an attractive approach to treat high-risk HB, especially when combined with the standard-of-care therapeutic cisplatin.

## Graphical abstract

**Figure:**
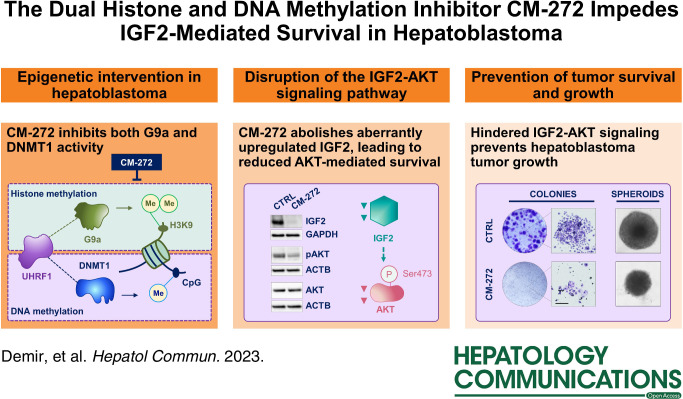

## INTRODUCTION

Hepatoblastoma (HB) is the most frequent primary liver tumor in childhood, representing more than 80% of all pediatric malignant liver tumors.^1^ Although most HB cases occur sporadically, cancer predisposition syndromes such as familial adenomatous polyposis coli and Beckwith-Wiedemann syndrome have been associated with HB pathogenesis,^2^,^3^ being linked either with germline mutation of the adenomatous polyposis coli gene or epimutations at the IGF2 locus. Approximately 70% of the sporadic HB cases harbor beta-catenin (CTNNB1) gene mutations as the main driving event,^4^ which leads to constitutive activation of the Wnt signaling pathway. Nonetheless, HB tumors can be considered genetically speaking as relatively simple, as they harbor only three mutations per tumor genome on average.^5^,^6^ As HB presents in different histological forms and more importantly with varying clinical outcomes, which cannot be explained by genetics alone, the contribution of additional underlying molecular mechanisms, such as epigenetic alterations, are being increasingly discussed.^7^,^8^ One prominent epigenetic alteration of HB tumors is the transcriptional upregulation of IGF2 due to changes in the DNA methylation level at its imprinting control region, thereby leading to activation of the phosphatidylinositol-3-kinase (PI3K)-AKT survival pathway.^9^,^10^ Notably, PI3K-AKT signaling has been implicated in the resistance to several types of anticancer regimens.^11^,^12^

The treatment of HB has dramatically improved over the last few decades due to advancements in surgical procedures and refinements in clinical risk stratification, yet there is still an unmet need for improved treatment options for patients with refractory disease.^13^ Survival properties of the tumor that are often caused by epigenetic alterations^14^ are anticipated to be responsible for individual differences in chemosensitivity. Thus, the use of epigenetic drugs to reverse defective expression of genes involved in resistance processes is an attractive approach to convert a chemoresistant tumor into a chemosensitive one. Histone deacetylase inhibitors are known to exert substantial anticancer activity by selectively inducing transcriptional reprogramming through complex mechanisms impacting on post-translational modifications of histone proteins.^15^ However, drug resistance is a major concern for the use of HDA histone deacetylase C inhibitors in anticancer therapies.^16^ Among various potential mechanisms, DNA methyltransferase (DNMT)-mediated DNA hypermethylation has been discussed as one cause underlying resistance to histone deacetylase inhibition.^17^ Thus, pharmacological cotargeting of DNA methylation and histone modifications could be a promising strategy to overcome drug resistance. Indeed, first trials have shown clinical benefit for the treatment of hematologic malignancies by using the 2 epigenetic-modifying drugs decitabine and vorinostat with chemotherapy.^18^,^19^

The epigenetic reader UHRF1 (ubiquitin like with PHD and RING finger domains 1) is an oncogene that is highly expressed in several malignancies,^20^ including HB and HCC.^21^,^22^ It functions as a key master for connecting DNA methylation to histone modifications.^23^ On one side, UHRF1 recruits DNMT1 to hemimethylated DNA, thus maintaining epigenetic modifications left on the newly synthesized DNA strand during cell division.^24^ On the other side, UHRF1 recognizes G9a-established trimethylated histone H3 at lysine 9 (H3K9me3), thereby facilitating the localization of H3K9me3 to pericentric heterochromatin.^25^ Due to its potential to interact with both hemimethylated DNA and H3K9me3, UHRF1 has an important role in both DNA and histone methylation during mitotic cell division.^26^ Hence, inhibition of UHRF1 serves as an appealing new anticancer strategy.

In this study, we determined the clinical relevance of repressing the epigenetic footprint of UHRF1 in patient-derived xenograft (PDX) models by using CM-272, a dual small molecule inhibitor targeting G9a and DNMT1 activity.^27^

## METHODS

### Patient data

The RNA expression data sets Ikeda-67-GSE131329 (https://www.sciencerepository.org/european-journal-of-molecular-cancer) and Carrillo-Reixach-66-GSE133039^28^ were retrieved from the R2 genomics analysis and visualization platform (https://hgserver1.amc.nl/). Genes were normalized to the housekeeping gene TATA-box-binding-protein and depicted as relative gene expression. Gene expression data and 5-year survival data of 365 patients with HCC were retrieved from Human Protein Atlas database v22.0 (https://www.proteinatlas.org/) and used for survival analysis.

### Cell lines

We used the HB cell lines HepG2 (American Type Culture Collection, Manassas, VA) and HepT1 (from Dr. T. Pietsch), the HCC cell lines Hep3B (American Type Culture Collection) and HUH7 (Japanese Collection of Research Bioresources, Osaka, Japan), the HB cell lines PDX214, PDX243, PDX282, PDX295, PDX303, PDX344, and PDX364 developed from PDX transplantation models (XenTech, Evry, France), and primary adult (HDFa) and neonatal (HDFn) human dermal fibroblasts (both American Type Culture Collection). Established cell lines were cultured in RPMI 1640 supplemented with 10% fetal bovine serum and 1% penicillin/streptomycin (all from Thermo Fisher, Waltham, MA), PDX cell lines in advanced DMEM/F12 containing 10 mg/L insulin supplemented with 10% fetal bovine serum, 1% penicillin/streptomycin, 1% l-glutamine (all from Thermo Fisher), and 20 µM Y-27632 (Selleckchem, Chesterbrook, PA) at 37°C in 5% CO_2_.

### Viability assay

Cells were seeded as 5×10^4^ cells/well in 96-well plates and, after 24 hours, exposed to 10 increasing concentrations of cisplatin, doxorubicin, CM-272, decitabine (5-azacytidine) (all from Selleckchem) and BIX-01294 trihydrochlorid hydrate (Sigma-Aldrich) ranging from 5 nM to 100 µM with DMSO. Rescue experiments on CM-272 using 10 ng/mL recombinant human IGF2 (RD Systems, Minneapolis, MN) were done for 48 hours by refreshing media after 24 hours. Following 48 hours of incubation, cell viability was measured using MTT (3-(4, 5-dimethylthiazol-2-yl)-2, 5-diphenyltetrazolium bromide) assays and absorbance measurement as described.^21^ Half-maximal inhibitory concentrations (IC50) and AUC values were calculated by plotting nonlinear regression of drug response curves using GraphPad Prism 8 (GraphPad Software, San Diego, CA).

### Synergy assay

Synergy of pairwise drug combinations was investigated by using MTT-based cell viability data of tumor cells treated with cisplatin, doxorubicin, and CM-272 for 48 hours. Synergy scores were determined by Combenefit software^29^ applying the highest single-agent statistical reference model.

### Proliferation assay

Proliferation was detected by using Click-iT^tm^ EdU cell proliferation kit (Thermo Fisher), according to the manufacturer’s instructions. A total of 2×10^5^ cells were seeded in a 24-well plate 24 hours prior to labeling with 100 µM ethynyl deoxyuridine and subsequent exposure to 200 nM CM-272 or DMSO for another 24 hours. Then, cells stained with Alexa Fluor 555 azide and Hoechst 33342 counterstain were captured with the EVOS M7000 imaging system (Thermo Fisher) and ethynyl deoxyuridine–positive nuclei counted in relation to Hoechst 33342-positive nuclei.

### Apoptosis assay

Apoptotic cell count was detected by CellEvent™ Caspase-3/7 Green Detection Reagent (Thermo Fisher) according to the manufacturer’s instructions. A total of 2×10^5^ cells/well were seeded in a 24-well plate, and following 24 hours of exposure to 100 nM CM-272 or DMSO, cells positive for active caspase 3 and 7 acquired with EVOS M7000 (Thermo Fisher) were counted.

### Colony formation assay

A total of 2×10^3^ cells/well were seeded in 6-well plates and incubated for 48 hours at 37°C. Then, cells were exposed to either DMSO or 100 nM CM-272 for 10 days. On formation of colonies, cells were stained with 0.5% crystal violet (Sigma-Aldrich) in 20% methanol for 2 hours. Pictures of colonies were taken by GelJet Imager and EVOS M7000 (Thermo Fisher) and counted.

### Spheroid assay

Cells were seeded at a density of 1×10^3^ cells/well into ultra-low attachment round-bottom 96-well plates (Corning, Corning, NY) for 5 days and established spheroids were exposed to 100 nM CM-272 or DMSO control. Spheroid images were captured at days 0, 2, and 6 with EVOS M7000 (Thermo Fisher), and spheroid volumes were calculated, as described.^30^

### siRNA-mediated knock-down

Knock-down experiments were conducted using SMARTpool siRNAs (Dharmacon Corporation, Lafayette, CO) for IGF2 (M-004093-01) and nontargeting control (D-0011210-01-05). In brief, 100 µL of siRNA and 5 µL lipofectamine 2000 were individually mixed with 250 µL serum-free medium in separate tubes and incubated for 5 minutes at room temperature. Then, the 2 mixtures were combined by gentle pipetting and incubated for 20 minutes at room temperature. The siRNA-lipofectamine mixture was then added to 1×10^6^ cells and incubated in 2 mL culturing medium in 6-well plates for 48 hours.

### Western blot analysis

Whole-cell lysates were extracted in 30 mM TrisHCl/150 mM NaCl/1% Triton-X/10% glycerol. Nuclear extracts were isolated in 10 mM Hepes/2.5 mM KCl/1.5 mM MgCl_2_/1 mM DTT/0.1 mM EDTA/0.625% Igepal, followed by a second lysis in 20 mM Hepes/0.4 mM NaCl/1.5 mM MgCl_2_/0.5 mM DTT/0.05 mM ETDA/25% glycerol. Proteins were denatured, separated in gradient gel and transferred to nitrocellulose membranes as described.^21^ Membranes were incubated overnight at 4°C in anti-H3K9me2 (Active Motive, Waterloo, Belgium), anti-IGF2 (RD Systems), anti-AKT or anti-pAKT-ser473 antibody (both Cell Signaling Technologies, Danvers, MA) in 1:1000 dilution. Anti-beta-actin, anti-lamin B1 (both Cell Signaling Technologies) and anti-glyceraldehyde-3-phosphate dehydrogenase (Thermo Fisher) in a 1:10,000 dilution served as a loading control. Following the incubation with a secondary goat-anti-rabbit HRP antibody (Dako Denmark, Glostrup, Denmark) in a 1:5000 dilution, protein bands were detected by enhanced chemoluminescence reagent (Amersham Biosciences, Amersham, UK) using the ChemiDoc XRS+ imaging system (Bio-Rad). All antibody dilutions were prepared in 5% milk in PBS-T.

### Pyrosequencing

DNA extraction and bisulfite-convertion were performed as described.^21^ A PCR reaction specific for LINE-1 elements^31^ was then performed using 200 ng bisulfite-treated DNA, 0.2 U hot start GoTaq G2 polymerase (Promega, Madison, WI), 5× GoTaq buffer, 2 mM dNTPs, 1.5 mM MgCl_2_, and 500 nM of the primers LINE1-F (TTTTGAGTTAGGTGTGGGA) and biotinylated LINE1-R (AAAATCAAAAAATTCCCTTTC). PCR reactions were run at 95°C for 4 minutes and 45 cycles of 95°C for 45 seconds, 57°C for 45 seconds, and 72°C for 60 seconds. Then, PCR products were sequenced with the AGTTAGGTGTGGGATATAGT sequencing primer, applying PyroMark Gold Q24 reagents (Qiagen) according to the manufacturer’s instructions.

### RNA sequencing

Total RNA was isolated from PDX282 and PDX303 and libraries prepared and sequenced as described.^32^ Split-read alignment against the human genome assembly hg19 (GRCh37) and UCSC known gene annotation was accomplished using STAR aligner v2.4.2a.^33^ Number of reads mapping to annotated genes was quantified using HTseq-count v0.6.0^34^ and read counts were normalized and analyzed for differentially expressed genes by using the Bioconductor package DESeq2.^35^ Identification of enriched pathways by Kyoto Encyclopedia of Genes and Genomes (KEGG) pathway analysis was carried out with differentially expressed genes >2-fold with a *p*-value<0.05 as an input via WEB-based GEne SeT AnaLysis Toolkit (https://www.webgestalt.org/) and clustergrams highlighting up- or downregulated genes were obtained from Enrichr database (https://maayanlab.cloud/Enrichr/). Protein-protein interaction network map of differentially expressed genes was generated by STRING web tool v11.5 (https://string-db.org/).

### Animal studies

The preclinical study in mice was carried out by XenTech, as described,^36^ under the license for experiments on vertebrate animals (APAFIS#29136-2020121415204532). In brief, cryopreserved tumor pieces of the PDX model HB-282 were subcutaneously implanted in the back of 5-week-old female nude-Foxn1nu mice (ENVIGO, Gannat, France) and grown until a volume of approximately 100 mm^3^ was reached. Mice were then treated with either vehicle or 5 mg/kg CM-272 in 10% DMSO/40% PEG300/5% tween/45% NaCl by i.p. injection for 5 days on and 2 days off. Tumor volume was calculated as described.^30^ All animals were weighed at tumor measurement times and monitored every day for physical appearance, behavior, and clinical changes.

### Statistical analyses

Statistical analyses were performed with GraphPad Prism 8 (GraphPad Software). Data were visualized as mean±SEM or SD. For all assays, differences between the 2 groups were analyzed using Student *t* test. For the survival analysis, Kaplan-Meier method and log-rank tests were performed. Pearson method was applied for deducing the correlation between the 2 groups by using two-tailed analysis of confidence intervals of 0.95. Calculation of the band densities of western blot analysis was done using ImageJ v1.54b image processing tool. Values of *p*<0.05 were considered significant for all analyses.

## RESULTS

### DNMT1, EHMT2, and UHRF1 genes are associated with poor prognosis in liver cancer

**FIGURE 1:**
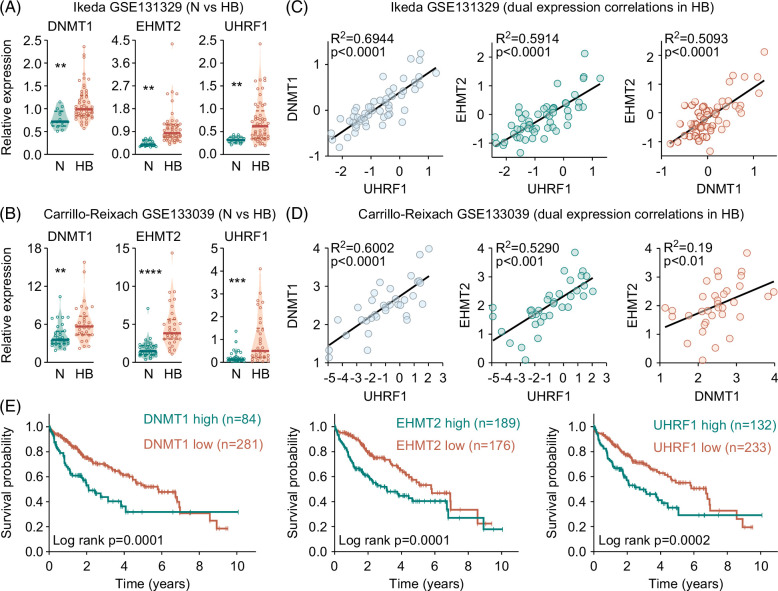
DNMT1, EHMT2, and UHRF1 genes are associated with a poor prognosis in liver cancer. (A, B) RNA expression levels of DNMT1, EHMT2, and UHRF1 genes in normal liver (N) and HB. Fragments per kilobase of transcript per million mapped reads of candidate genes were retrieved from the data sets (A) Ikeda GSE131329 (N=14, HB=53) and (B) Carrillo-Reixach GSE133039 (N=33, HB=33) and normalized to the housekeeping gene TATA-box-binding-protein. The violin plot displays individual values (circles), the median (solid lines) and the 25/75th percentiles (dashed lines) of the group. Student *t* test was applied to calculate significances, with **p<0.01, ***p<0.001, ****p<0.0001. (C, D) Correlation of gene expression between DNMT1, EHMT2, and UHRF1 in the (C) Ikeda GSE131329 and (D) Carrillo-Reixach GSE133039 data sets. Two-tailed Pearson test was performed, and correlation coefficients were calculated. The linear regressions are given as solid black lines. (E) Kaplan-Meier curves displaying survival probabilities for patients with HCC (n=365) retrieved from the Human Protein Atlas database with either high or low *DNMT1*, EHMT2, and UHRF1 expression. The log-rank Mantel-Cox test was used to calculate significance. Abbreviations: DNMT1, DNA methyltransferase1; EHMT2, *euchromatic histone lysine methyltransferase 2*; HB, hepatoblastoma; UHRF1, *ubiquitin like with PHD and ring finger domains 1*.

In order to investigate the expression levels of the epigenetic regulators DNMT1 and G9a (encoded by the gene *euchromatic histone lysine methyltransferase 2*, EHMT2), as well as their recruiting factor UHRF1, we retrieved data from 2 comprehensive and publicly available data sets on HB from the R2 database. We observed significantly higher expression levels of the DNMT1, EHMT2, and UHRF1 genes in HB than in normal liver in both data sets (Figure 1A, B). Interestingly, levels of DNMT1, UHRF1, and EHMT2 expression are highly correlated with each other in patients with HB (Figure 1C, D).

Next, we wanted to evaluate the survival probability of liver cancer patients with high-expression and low-expression of DNMT1, EHMT2, and UHRF1 genes. Due to the lack of available survival data from patients with HB, we examined data from patients with HCC. Kaplan-Meier curves revealed significant differences between patients with high-expression and low-expression of DNMT1, EHMT2, and UHRF1 (Figure 1E), with 5-year survival rates of 32%, 41%, and 35% compared to 53%, 56%, and 55%, respectively. In conclusion, our *in silico* analysis revealed a high concomitant expression of the epigenetic genes DNMT1, EHMT2, and UHRF1 in liver cancer patients with inferior outcomes.

### Dual inhibition of DNTM1 and G9a by CM-272 effectively reduces the growth of liver cancer cells

**FIGURE 2:**
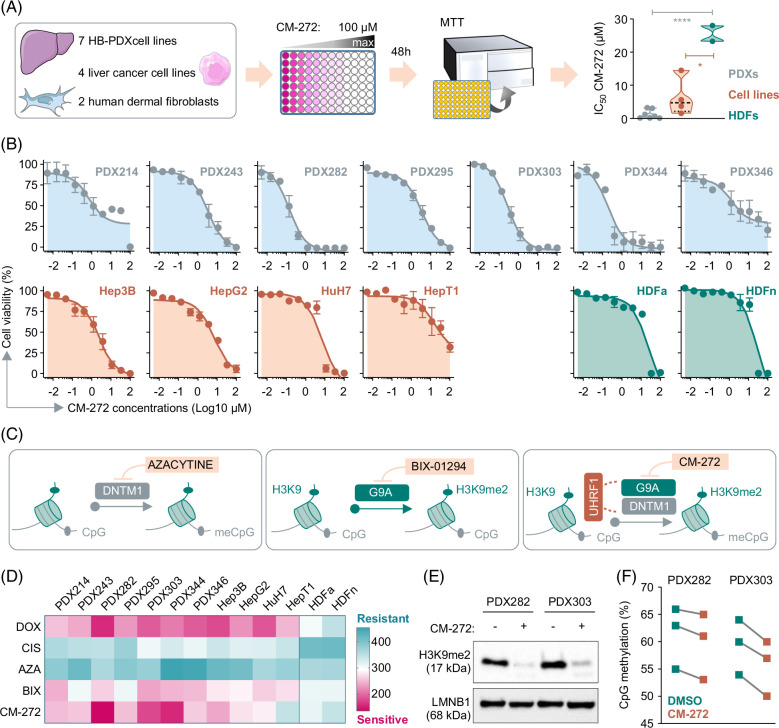
Dual inhibition of DNTM1 and G9A effectively reduces growth of liver cancer cells. (A) Schematic overview of the in vitro drug testing platform comprising four liver cancer cell lines, seven HB PDX cell lines, and normal HDFs. The graph on the right depicts IC50 values of CM-272–treated cell lines, showing individual values (circles), the median (dashed lines) and the 25/75th percentiles (dotted lines) of the group. Student *t* test was applied to calculate significances, with *p<0.05, ****p<0.0001. (B) Dose-dependent drug response curves of cell lines on exposure to 10 increasing CM-272 concentrations ranging from 5 to 100 µM. All data points represent 2 independent experiments with duplicate measurements, and the error bars represent±SEM. (C) Schematic illustrations depicting the inhibitory mechanisms of DNA methylation by 5-azacytidine, histone 3 lysine 9 methylation (H3K9me) by BIX-0194, and dual inhibition of DNMT1 and G9a by CM-272. (D) Heatmap showing drug sensitivity of cell lines to DOX, CIS, AZA, BIX, and CM-272. AUC values represent the mean of 2 independent cell viability experiments, each consisting of duplicate measurements. The color scale from blue to pink indicates increasing sensitivity to drug treatment. (E) Western blot detection of H3K9me2 in PDX282 and PDX303 tumor cells on 16 hours exposure to 100 nM CM-272. LMNB1 was used as a nuclear loading control. (F) Pyrosequencing results showing the global methylation levels at 3 CpG dinucleotides of LINE-1 elements of PDX282 and PDX303 cells following 100 nM CM-272 exposure for 16 hours. Abbreviations: AZA, 5-azacytidine; BIX, BIX-01294; CIS, cisplatin; DNMT1, DNA methyltransferase1; DOX, doxorubicin; HB, hepatoblastoma; HDF, human dermal fibroblast; HDFa, adult human dermal fibroblast; HDFn, neonatal human dermal fibroblast; H3K9me2, dimethylated histone 3 serine 9; LMNB1, lamin B1; PDX, patient-derived xenograft.

To determine the impact of the simultaneous inhibition of DNTM1 and G9A through the dual inhibitor CM-272^27^ on liver tumor cells, we made use of a drug testing platform consisting of 4 conventional liver cancer cell lines, 7 previously established patient-derived HB xenograft cell lines, and 2 human dermal fibroblast cell lines as noncancerous controls. MTT-based viability assays after CM-272 exposure demonstrated significantly lower median half-maximal inhibitory concentrations (IC50) for HB PDXs (0.7 µM) and liver cancer cell lines (6.4 µM) in comparison to healthy fibroblast controls HDFa (23 µM) and HDFn (28 µM) (Figure 2A). AUC values calculated from drug-response curves to 10 increasing concentrations of CM-272 further proved the high sensitivity of PDX-derived (mean AUC: 213.2) and conventional liver cancer cell lines (mean AUC: 284.7) toward CM-272, while healthy controls HDFa (312.3) and HDFn (312.3) remained nonresponsive (Figure 2B), suggesting the selective and noncytotoxic targeting properties of CM-272.

In order to see if the tumor cell-impacting effect of the dual epigenetic inhibitor CM-272 can also be reached by inhibiting just one epigenetic mechanism, being either DNA demethylation or histone methylation (Figure 2C), we applied the demethylating agent 5-azacytidine as well as the specific inhibitor of G9a histone methyltransferase BIX-01294 to our drug testing platform. Exposing the liver tumor cells to 5-azacytidine, BIX-01294 and CM-272 in a similar 10-point dose-response curve setting revealed a much higher efficacy of CM-272 on tumor cell survival (mean AUC: 239.2) in comparison to 5-azacytidine (mean AUC: 399.4) and BIX-01294 (mean AUC: 268.5) (Figure 2D). Moreover, whereas all models demonstrated a minor response to cisplatin, the standard-of-care therapy for patients with standard-risk HB,^37^ CM-272 treatment led to a drastic decrease in cell viability (Figure 2D), similar to the one toward doxorubicin, which is routinely used for patients with high-risk HB^38^ (Figure 2D).

Furthermore, we investigated the on-target effects of CM-272 treatment in 2 selected PDX cell lines. Immunoblotting showed a decrease in the total levels of histone H3 lysine 9 dimethylation (H3K9me2) by CM-272, demonstrating the successful inhibition of the G9a enzyme (Figure 2E). Quantification of the global DNA methylation by pyrosequencing LINE-1 elements in the tumor genome revealed a decline of CpG methylation levels when CM-272 was present, suggesting the hindered function of DNMT1 (Figure 2F).

### CM-272 causes transcriptional downregulation of IGF2

**FIGURE 3:**
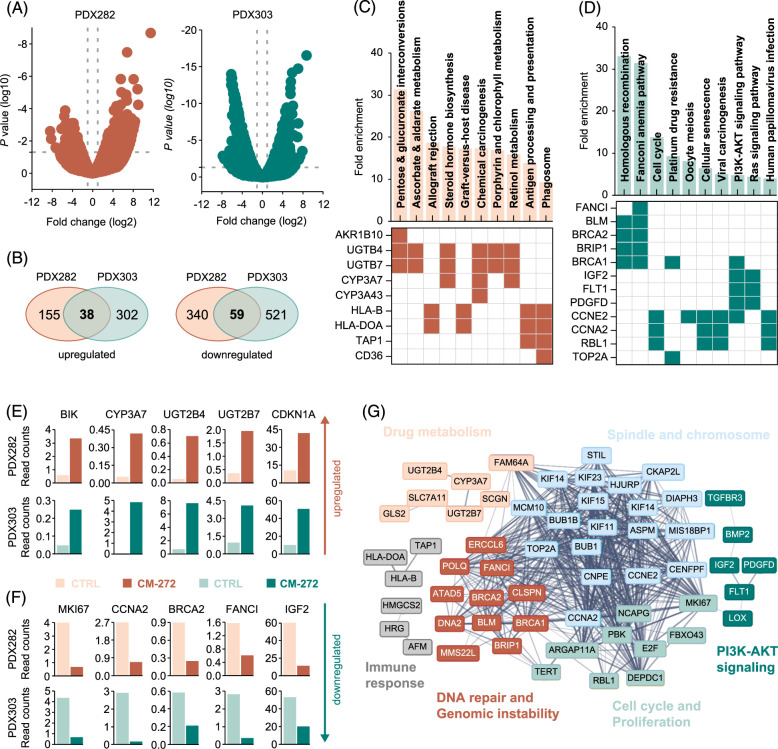
Transcriptional consequences of CM-272 treatment in liver cancer cells. (A) Volcano plots of differentially expressed genes in PDX282 and PDX303 cells on 16 hours of exposure to 100 nM CM-272, as determined by RNA sequencing. The log10-transformed *p*-values are plotted against the average log2 fold changes in gene expression, with dashed lines indicating *p*<0.05 and fold changes >2. (B) Venn diagrams summarizing the numbers of upregulated and downregulated expressed genes for PDX282 and PDX303. (C, D) KEGG pathway analysis (upper) and clustergrams (lower) for commonly (C) upregulated and (D) downregulated genes of PDX282 and PDX303, revealing enrichment in cellular and molecular pathways and associated key genes. (E, F) Bar graphs representing RNA expression of the top-scoring candidate genes of the enriched pathways in the DMSO- (light) and CM-272- (dark) treated PDX282 (orange) and PDX303 (green) cells. (G) Protein-protein interaction network map visualizing CM-272-regulated candidate genes. The nodes and edges represent query genes and predicted relations between candidates, respectively. The thickness of the lines indicates the strength of data support and stronger interaction among the query proteins. Abbreviations: BIK, BCL2-interacting killing; BRCA2, breast cancer gene 2; CCNA2, cyclin 2A; FANCI, Fanconi anemia complementation group I; KEGG, Kyoto Encyclopedia of Genes and Genomes; PDX, patient-derived xenograft; PI3K, phosphatidylinositol-3-kinase.

In order to obtain a mechanistic understanding of the CM-272 treatment on HB, we performed RNA sequencing of PDX282 and PDX303 cells exposed to CM-272. Comparing the global transcriptional expression levels of CM-272–treated tumor cells with DMSO-treated controls revealed a considerable number of significantly (*p*<0.05) altered (>2-fold) genes (Figure 3A), thereby underscoring the pleiotropic effect of simultaneously inhibiting DNA and histone methylation. Strikingly, we found an overlap of 38 upregulated and 59 downregulated genes that are common in both PDX282 and PDX303 models (Figure 2B).

To further investigate the functional impacts of CM-272 treatment on cellular and molecular processes, we applied KEGG pathway enrichment analysis to the differentially expressed genes common to both models (Figure 3C-D). Clustergrams demonstrated KEGG terms for the metabolism of various molecules, such as pentose, glucose, ascorbate, aldarate, steroid, porphyrin, and retinol to be highly enriched in the upregulated genes (Figure 3C). This enrichment was mainly based on elevated RNA levels of the genes coding for the cytochrome P450 3A enzyme subfamily member CYP3A7, as well as the UDP glucuronosyltransferase 2 family polypeptide B4 subfamily proteins UGT2B4 and UGT2B7 (Figure 3E). These 2 enzyme families are known to play essential roles in drug detoxification and the conversion of xenobiotics into biological substrates. Moreover, we found the proapoptotic protein BCL2-interacting killing and the cyclin-dependent kinase inhibitor 1A, a tumor suppressor gene that is negatively regulated by UHRF1,^39^ to be upregulated in the presence of CM-272 (Figure 3E).

For the downregulated genes, we detected a strong enrichment of KEGG terms for homologous recombination, DNA repair, cell cycle, and PI3K-AKT signaling (Figure 3D), which was mainly evoked by a significant decrease in transcript levels of the Fanconi anemia complementation group I, breast cancer gene 2, cyclin 2A, and IGF2 (Figure 3F). In addition, we found inhibition of the proliferation marker Ki-67 on CM-272 treatment. The generation of a protein-protein interaction network map with the differentially expressed genes underscored the close relationship between the molecular groups identified by KEGG pathway enrichment analysis (Figure 3G). Collectively, these data suggest that CM-272 induces drug metabolic processes while impeding DNA integrity, tumor growth, and most importantly, IGF2-associated PI3K-AKT signaling of HB tumor cells.

### CM-272 inhibits liver cancer cell growth by impeding PI3K-AKT signaling

**FIGURE 4:**
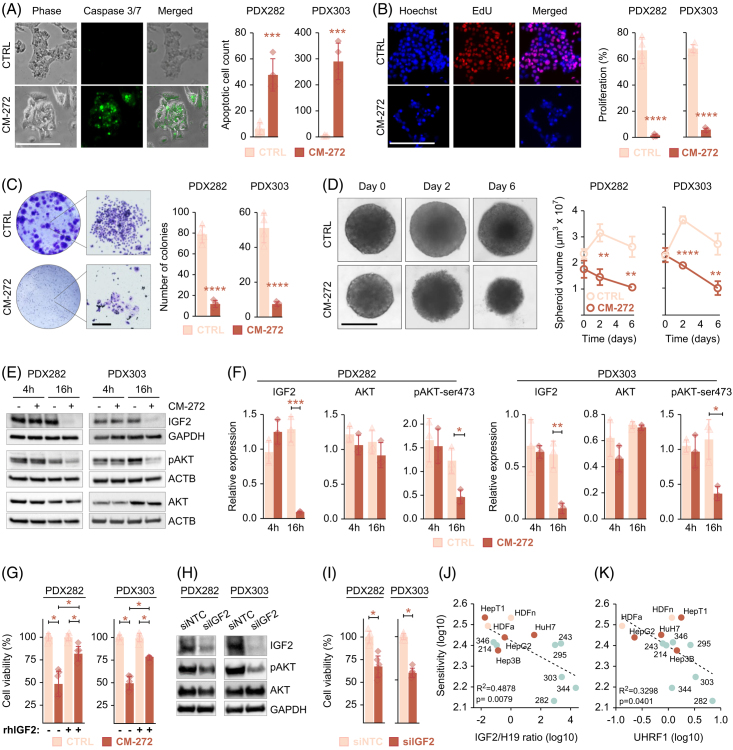
Cellular consequences of CM-272 treatment in liver cancer cells. (A) Apoptotic cells were detected in PDX282 and PDX303 cells, which were exposed to DMSO (CTRL) or 200 nM CM-272 for 48 hours by staining active caspase 3/7 substrate (green) and quantified in relation to adherent cells (phase contrast). The bar graphs represent 2 independent experiments with 6 replicate measurements. (B) Proliferating cells were detected by Click-it EdU-staining (red) and quantified in relation to Hoechst 33342-stained nuclei (blue). PDX282 and PDX303 cells were exposed to DMSO (CTRL) or 200 nM CM-272 for 48 hours. The bar graphs represent 2 independent experiments with 6 replicate measurements. (C) Long-term growth of the PDX cells was monitored by colony formation assay. Cells were exposed to DMSO (CTRL) or 50 nM CM-272 for 10 days, and colonies were stained with crystal violet. Representative wells (left) and magnified views (right) are displayed. The bar graphs represent two independent experiments with two replicate measurements. (D) Established tumor spheroids of PDX282 and PDX303 cells were treated with DMSO (CTRL) or 200 nM CM-272 for 6 days. The line graph represents mean spheroid volumes of one experiment consisting of triplicate measurements. (E) Western blot detection of IGF2, AKT and phosphorylated AKT (pAKT) levels in PDX282 and PDX303 liver tumor cells on exposure to 100 nM CM-272 for 16 hours. ACTB or GAPDH were used as loading control. (F) The bar graphs represent densitometrical quantifications of protein amounts detected by western blot of 3 independent experiments. (G) Sensitivity of HB PDXs toward CM-272 detected as cell viability in the presence (+) or absence (−) of 10 ng/mL rhIGF2 for 48 hours. The bar graphs represent 2 independent experiments with 3 replicates. (H) Western blot detection of IGF2, pAKT, and AKT levels after 48 hours of transfection with siIGF2 or siNTC. (I) Viability of tumor cells following IGF2 knock-down for 48 hours. The bars respresent 2 independent experiments with 3 replicates. (A–I) All error bars represent±SD. *p*-values were calculated using a two-tailed unpaired Student *t* test, with *p<0.05, **p<0.01, ***p<0.001, ****p<0.0001. All scale bars represent 200 μm. (J) Correlation of the log10-transformed IGF2/H19 ratios or (K) UHRF1 expression (RNAseq counts normalized to TATA-box-binding-protein) of cell lines with the sensitivity against CM-272 treatment (log10-transformed AUC values). Two-tailed Pearson test was performed, and correlation coefficients were calculated. The linear regression is given as a dashed line. Abbreviations: ACTB, beta-actin; CTRL, control; EdU, ethynyl deoxyuridine; GAPDH, glyceraldehyde-3-phosphate dehydrogenase; HDFa, adult human dermal fibroblast; HDFn, neonatal human dermal fibroblast; pAKT, phosphorylated AKT; PDX, patient-derived xenograft; rhIGF2, recombinant human IGF2; siIGF2, siRNA against IGF2; siNTC, nontargeting control; UHRF1, *ubiquitin like with PHD and ring finger domains 1*.

As the RNA sequencing data suggested induction of apoptosis, inhibition of growth and IGF2-mediated survival as key targets of CM-272, we explored the consequences of CM-272 treatment on the cellular behavior of HB tumor cells. In line with the elevated BCL2-interacting killing transcript levels, caspase 3/7 staining of CM-272–treated HB cells showed a significant increase of apoptotic cells, suggesting a subsequent induction of programmed cell death (Figure 4A). Consistent with the reduced cyclin 2A and marker Ki-67 transcript levels, proliferating cells were significantly reduced on CM-272 exposure in comparison to control cells (Figure 4B). In addition, the colony formation assay clearly revealed that the proliferative integrity of tumor cells was dramatically reduced in long-term cultures, as the CM-272–treated PDX cells formed only a few small colonies (Figure 4C). Moreover, we inspected the effect of CM-272 on established tumor spheroids, considering that three-dimensional cell cultures serve as better near-patient models. We detected that CM-272 treatment resulted not only in the repression of tumor growth, but also in a significant reduction of spheroid volume, while untreated spheroids maintained their ability to enlarge in volume (Figure 4D).

Of most interest, we wanted to validate the functional consequences of the downregulation of IGF2 expression by analyzing the PI3K-AKT signaling pathway, which is driven by IGF2. Western blot analysis of CM-272–treated PDX cells revealed a significant decrease of IGF2 protein levels and subsequently of AKT serine 473 phosphorylation, a constitutive phosphorylation site for PI3K-AKT pathway activation, while leaving the total amount of AKT protein unchanged (Figure 4E and F). However, we are not sure how IGF2 activity is transferred into the cells, as both receptors known to bind IGF2, namely IGF1 receptor and insulin receptor are highly expressed in our RNAseq data (data not shown). In order to investigate whether CM-272-induced cell death is indeed mediated by IGF2, we repeated our experiments in the presence or absence of recombinant human IGF2 (rhIGF2). Viability assays clearly indicated that the response of HB PDX cells to CM-272 exposure is significantly reduced when cells were simultaneously incubated with rhIGF2 (Figure 4G). Moreover, by introducing a siRNA-mediated knock-down of IGF2 in PDX cells, we could demonstrate a robust decrease of AKT activation while total AKT remained unchanged (Figure 4H), thereby leading to a significant decrease of the viability of IGF2-suppressed HB cells compared to the nontargeting control (Figure 4I). Having demonstrated that IGF2 is an important target of CM-272, we looked at the RNA levels of IGF2 and the reciprocally expressed H19 gene and compared the ratio of both genes to the drug response against CM-272. We found a clear correlation between high IGF2/H19 ratios and sensitivity to CM-272, thereby suggesting that high IGF2-expressing tumors, such as most HB tumors, are prone to dual epigenetic inhibitors (Figure 4J). Of interest, only UHRF1 expression, but not DNMT1 and EHMT2, was also correlated with sensitivity to CM-272 (Figure 4K). Collectively, our results clearly demonstrate that CM-272 inhibits the growth of HB cells and consequently triggers apoptosis by disrupting the PI3K-AKT survival pathway through downregulating IGF2.

### CM-272 synergizes with cisplatin in inducing cell death

**FIGURE 5:**
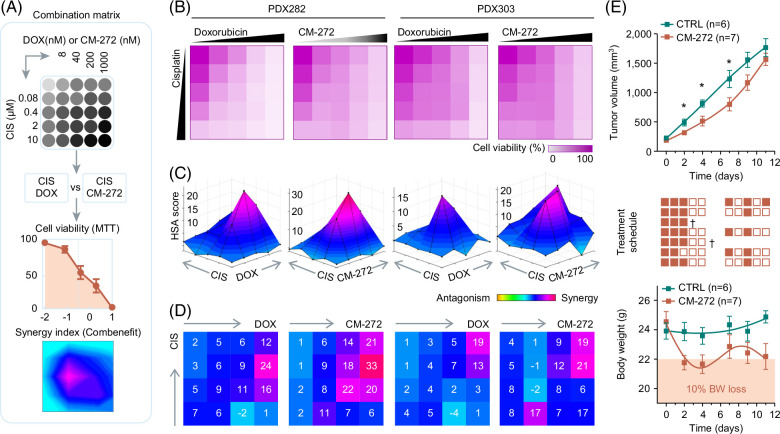
Preclinical testing of CM-272 in combination with cisplatin and *in vivo*. (A) Experimental set-up of combination experiments and synergy calculation. The two-drug combination matrix demonstrates the 4 increasing concentrations for CIS given in micromolar, and for DOX and CM-272 given in nanomolar range. (B) Heatmaps displaying the proportions of cell viability in PDX282 and PDX303 cells on exposure toward increasing concentrations of CIS, DOX, and CM-272 as two-drug combination matrix format (n=2, duplicate measurements). (C) Three-dimensional synergy landscapes shown separately for each two-drug combination of CIS and DOX as well as CIS and CM-272. Synergy was calculated with the Combenefit tool, and synergy scores obtained by using the highest single-agent statistic model. Areas highlighted in pink-purple represent high synergy, and dark blue for moderate synergy (n=2, duplicate measurements). (D) Synergy indexes demonstrate the synergy scores for every possible combination of two-drug concentrations. (E) Tumor growth (upper), treatment schedule (middle panel, with filled boxes indicating applied doses, empty boxes skipped doses and crosses death of animals), and body weight changes (lower panel) in mice treated with either CM-272 (n=7) or vehicle (n=6). Values correspond to mean tumor volumes and mean body weights±SEM. Shaded area indicates body weight loss of >10% of starting values. Abbreviations: CIS, cisplatin; CTRL, control; DOX, doxorubicin; MTT, 3-(4, 5-dimethylthiazol-2-yl)-2, 5-diphenyltetrazolium bromide; PDX, patient-derived xenograft.

To investigate the probable translation of CM-272 into future clinical application, we wanted to explore the possible beneficial impact of combining CM-272 with cisplatin, the backbone of HB therapy,^37^ on the suppression of tumor cell growth. To do so, we subjected the 2 models PDX282 and PDX303, with a high sensitivity toward CM-272 (IC50 138 and 266 nM, respectively) and high IGF2/H19 ratios (Figure 4J) to a 4×4-dose point combination matrix of CM-272 and cisplatin (Figure 5A). The combination of both drugs led to a striking reduction of cell viability, even at low nanomolar levels. This was highly comparable to the decrease observed in response to the combination of cisplatin and doxorubicin, the standard of care for patients with high-risk HB^38^ (Figure 5B). More importantly, depicting three-dimensional synergy landscapes and calculating synergy indexes for each drug combination revealed that CM-272 combined with cisplatin shows a stronger synergy than the clinically used cisplatin and doxorubicin combination (Figure 5C, D). Altogether, our findings clearly indicate that CM-272 highly synergizes with cisplatin for the inhibition of HB tumor cell growth, suggesting that CM-272 can be considered as a strong possible candidate for the treatment of patients with HB.

### CM-272 causes side effects in a HB PDX transplantation model

In order to provide a rationale for the clinical use, we tested CM-272 in a transplantation model of an established HB PDX^36^ using a dose of 5 mg/kg injected i.p. for 5 days per week, which has been shown to be effective and well tolerated in mice carrying HCC xenografts.^40^ After only 2 doses, we observed a strong reduction in tumor growth in CM-272-treated mice compared to vehicle-treated mice, which stayed significant until day 7 of treatment (Figure 5E). However, treatment had to be stopped after the third dose because of dramatically decreasing body weights in the CM-272–treated mice (Figure 5E). Moreover, 1 of the 7 mice in the CM-272 group died for unknown reasons at day 3 of treatment, and another mouse had to be sacrificed because of emaciated appearance and hunched posture on day 5. In those mice in whom the body weight subsequently recovered by day 7 after commencement of treatment, a reduced dosing scheme of 2.5 mg/kg CM-272 was given every second day. However, as tumor growth speeded up under this regimen and the mice lost body weight again, this preclinical trial was terminated on day 11. Of note, one mouse developed a heavily hemorrhagic tumor under treatment starting from day 5. In summary, these experiments indicate that CM-272 effectively inhibits tumor growth *in vivo*, but causes severe side effects at high doses.

## DISCUSSION

As genetics cannot explain the diverse course of HB,^5^,^6^ it has been proposed that transcriptional changes are at the root of the heterogeneous manifestation of this disease.^6^,^7^,^8^,^28^,^41^ Gene activity is mainly driven by epigenetic regulatory mechanisms such as DNA methylation and post-translational histone modifications, and their important roles have already been reported for HB in several studies.^7^,^8^,^10^,^21^ However, targeting the epigenetic machinery for therapeutic purposes is still not clinically achievable.

We have described in previous work on a small cohort of patients that the epigenetic reader UHRF1 is overexpressed in HB and associated with poor survival.^21^ By analyzing 2 large and recent transcriptomic data sets, we could corroborate our previous finding that UHRF1 is indeed a significantly overexpressed gene in patients with HB. Moreover, by applying the same data sets, we could also show a concomitant overexpression of the genes encoding DNMT1 and G9a, 2 interaction partners of UHRF1. Interestingly, the strong correlation of the three genes to each other clearly demonstrates that the UHRF1-DNMT1-G9a complex seem to play an important role in HB pathogenesis. This is in line with previous work on liver cancers, which showed simultaneous activation of the respective complex partners in HCC,^40^ cholangiocarcinoma,^42^ and just recently patients with HB.^43^ The clinical association between inferior patient survival and overexpression of the individual components of the UHRF1-DNMT1-G9a complex has been shown for many adult and pediatric cancers.^26^ Our previous work has also established that elevated levels of UHRF1 are predominantly observed in patients with HB with poor outcomes and the so-called C2 subtype of the 16-gene signature,^21^ which defines a more aggressive subtype of HB and is associated with poor prognosis.^41^ As no patient data are available for the 2 recent HB data sets used in this study, we performed survival analyses with a publicly available data set on HCC, a liver tumor that also occurs in pediatric patients and shares many molecular properties with HB.^44^ We could demonstrate an inferior overall survival of patients with HCC carrying high expressions of UHRF1, DNMT1, and G9a. Collectively, our data identified the UHRF1-DNMT1-G9a complex as an excessively activated and clinically relevant epigenetic regulator in patients with HB.

Chemical targeting of individual components of the UHRF1-DNMT1-G9a complex has already been investigated in liver cancers as a therapeutic strategy, such as the DNMT inhibitor decitabine^45^ and the G9a inhibitor BIX-01294,^46^ and showed only limited effects on cell viability when given as monotherapy. In contrast, dual targeting of both G9a and DNMT1 by the small molecule CM-272 has been reported to show significant therapeutic efficacy in hematological malignancies^27^ and most liver cancer types.^40^,^42^,^43^ In agreement with these data, our study demonstrates a significant reduction in tumor cell viability on CM-272 exposure in pediatric liver cancer cell lines and HB PDX models, while healthy cells remained nonresponsive toward this epigenetic perturbation. Most notably, we identified higher synergy scores when CM-272 was combined with cisplatin rather than with doxorubicin, even at low nanomolar concentrations, thereby suggesting that the combination of CM-272 and cisplatin could be an effective treatment for patients with HB. By investigating the transcriptional changes in HB tumor cells on CM-272 exposure, we have detected transcriptional downregulation of the survival factor IGF2 and subsequently reduced activity of PI3K-AKT signaling as the main target of the dual inhibition of UHRF1-driven consequences. This has been achieved by comprehensively profiling PDX-derived HB cell lines,^36^ which closely mirror the original tumors by expressing high levels of IFG2, as opposed to conventional HB cell lines grown on plastic for years that have been used in former studies.^21^,^43^ Moreover, our functional assays as well as RNA sequencing data in PDX-derived HB cell lines, highlighted that the transcriptional downregulation of IGF2 by CM-272 results in a reduced proliferation capacity of HB cells with decreased levels of the proliferation marker Ki-67 and a strong upregulation of apoptosis with increased levels of the proapoptotic protein BCL2 interacting killer. Constitutive activation of the PI3K-AKT pathway^9^ through epigenetic upregulation of IGF2^10^ and downregulation of IGF binding proteins^47^ is, along with the CTNNB1-mediated Wnt activation, the most important mechanism involved in HB development. It has been experimentally validated that IGF2 can act as the second survival factor for oncogene-induced tumorigenesis.^48^ Interestingly, depleting IGF2 from tumor cells has been described to render them more sensitive to several chemotherapeutic agents.^49^ In line with this, we found that HB models with high expression of IGF2 are most sensitive to CM-272 treatment, although low-dose insulin contained in the media might have prevented complete growth inhibition. Moreover, our rescue experiments clearly indicate that IGF2 is the major mediator of IGF2 activity, as the addition of recombinant IGF2 decreased sensitivity toward CM-272. Collectively, these data suggest that the dual inhibition of both G9a and DNMT1 by CM-272 is not only able to impede the IGF2-mediated survival of HB cells but might also be used to increase sensitivity to chemotherapy in order to help minimize short- and long-term side effects of drugs currently used for high-risk patients.^50^

Although mechanistically convincing, the preclinical testing of the dual inhibition of G9a and DNMT by CM-272 in HB-bearing mice unveiled some problems, which have to be considered before moving this drug further to the clinic. This was not anticipated, as a previous study showed no toxicity of CM-272 in healthy mice in terms of body weight loss as well as hematological and liver parameters when given for 4 weeks at a dose of 2.5 mg/kg body weight.^27^ A similar treatment schedule using doses of 2.5 and 5 mg/kg body weight corroborated the safety in leukemia and lymphoma models^27^ as well as pediatric and adult liver cancer models.^40^,^42^,^43^ However, our study recognized severe side effects, including sudden death, dramatic body weight loss, and tumor hemorrhage when CM-272 was used in the fast-growing and IGF2 high-expressing HB model PDX282.^36^ It might be speculated that targeting one of the driving forces of HB tumorigenesis, namely the constitutive activation of IGF2-PI3K-AKT survival signaling, which is not evident in most tumor models investigated so far,^27^,^40^,^42^,^43^ in concert with other as yet unknown HB-specific inherent alterations that are targeted by CM-272, could have resulted in an antitumor effect that induces a systemic response that cannot be tolerated *in vivo*.

In summary, we established the clinical relevance of the UHRF1-DNMT1-G9a complex in patients with HB and proved the antitumor effect of dually inhibiting DNMT1 and G9a activity in patient-derived xenograft HB models. Perturbing the epigenetic traces of UHRF1 led to the sustained inhibition of IGF2-PI3K-AKT–mediated survival of HB cells, both *in vitro* and *in vivo*. Although being highly synergistic with the standard-of-care medication cisplatin, CM-272 needs further safety optimizations before being considered for clinical use in patients with HB, particularly those with high IGF2 levels.
